# Microbial consumption of organophosphate esters in seawater under phosphorus limited conditions

**DOI:** 10.1038/s41598-018-36635-2

**Published:** 2019-01-18

**Authors:** Maria Vila-Costa, Marta Sebastián, Mariana Pizarro, Elena Cerro-Gálvez, Daniel Lundin, Josep M. Gasol, Jordi Dachs

**Affiliations:** 1 Department of Environmental Chemistry, IDAEA-CSIC-Jordi Girona 18-26, Barcelona, 08034 Barcelona, Catalunya Spain; 2 Departament de Biologia Marina i Oceanografia, Institut de Ciències del Mar, CSIC, Barcelona, Catalunya Spain; 3 Instituto de Oceanografía y Cambio Global, IOCAG, Universidad de Las Palmas de Gran Canaria, 35214 Gran Canaria, Spain; 4 Centre for Ecology and Evolution in Microbial Model Systems, EEMiS, Linnaeus University, Barlastgatan 11, 391 82 Kalmar, Sweden

## Abstract

The anthropogenic perturbation of the phosphorus (P) marine biogeochemical cycle due to synthetic organophosphorus compounds remains unexplored. The objective of this work was to investigate the microbial degradation of organophosphate triesters (OPEs), widely used as plasticizers and flame retardants, in seawater and their effects on the physiology and composition of microbial communities. Experiments were performed in July 2014 using surface seawater from the Blanes Bay Microbial Observatory (NW Mediterranean) to which OPEs were added at environmentally relevant concentrations. The concentrations of OPEs in the dissolved-phase generally decreased after 24 hours of incubation at *in situ* conditions. The fitted first order reaction constants were significantly different than zero for the trihaloalkyl phosphate, tris(2-chloroethyl) phosphate and trialyl phosphate tricresyl phosphate. In general, OPEs triggered an increase of the percentage of actively respiring bacteria, total bacterial activity, and the number of low-nucleic acid bacteria, and a decrease in the percentage of membrane-compromised bacteria. Members of some bacterial groups, in particular Flavobacteria, increased their specific activity, indicating that seawater contains bacteria with the potential to degrade OPEs. In aged seawater that was presumably depleted of labile dissolved organic carbon and inorganic P, alkaline phosphatase activities significantly decreased when OPEs were added, indicating a relief on P stress, consistent with the role of OPEs as potential P sources.

## Introduction

Phosphorus (P) is an essential nutrient for all living organisms, required for the synthesis of nucleic acids, cell membrane phospholipids as well as other key biological molecules. In many marine regions, P is often at suboptimal concentrations to support cell growth, thus limiting productivity^1^. This is particularly true in the Mediterranean Sea, the vast low-nutrient/low-chlorophyll zones in the central oceanic oligotrophic gyres, as well as coastal areas with high nitrogen loads^2–4^. Marine planktonic organisms have evolved strategies to cope with low and fluctuating P concentrations, including mechanisms of intracellular P storage, minimization of P requirements and P niche adaptations^5^. Moreover, during periods of low concentrations of the preferred form of P – phosphate (Pi) – microbes turn to the less energetically favorable dissolved organic phosphorus (DOP), which is present at larger concentrations than Pi^6^. DOP is mostly composed by phosphoesters and phosphonates, although specific components are still poorly characterized^5^.

During the last century, an increasing number of synthetic organic compounds containing P have been introduced into the environment. Examples of legacy organic pollutants used in the 20th century are organophosphorus pesticides, herbicides and chemical warfare agents, mainly esters and thiols. Some of them are currently under regulation^7^ due to their known acute and chronic toxicology^8^. Conversely, organophosphate triesters (OPEs) are emerging persistent organic pollutants (POPs) currently used as flame retardants and plasticizers in consumer products, partly due to the gradual ban of polybrominated diphenyl ethers (PBDEs) as flame retardants^9,10^. OPEs have suspected toxicological effects on living organisms, e.g. related to cancer, hormone disruption and neurotoxicity^11^. Little is known about their potential biodegradability in the marine environment, even though OPEs have been suggested to be a potential source of organic P in oligotrophic marine regions^12,13^ of a magnitude larger than the previously estimated atmospheric inputs of organic P^14^.

OPE flame retardants and plasticizers have been found in the ocean atmosphere, with a ubiquitous occurrence from the North and Mediterranean Seas to the global ocean^13,15–19^. Recently, OPEs have also been reported from surface waters in the North Atlantic and Arctic oceans^17^, and in the Yellow sea^20^. Although some models have predicted relatively short residence times of these compounds in the environment^21^, OPE ubiquity indicates that they are persistent enough to undergo long-range atmospheric and oceanic transport. This persistence, however, might be compound dependent, as OPEs comprise compounds with diverse chemical structures, subject to a number of different mechanisms and degree of degradation in the environment.

Microbial degradation of OPE flame retardants and plasticizers has been studied with isolates of the Alphaproteobacteria, *Sphingobium* sp. strain TCM1 and *Sphingomonas* sp. Strain TDK1^22–25^. Both strains could grow using OPEs as the sole source of P. The enzymes responsible for the degradation of OPEs were identified as haloalkylphophorus hydrolases, a set of phosphotriesterases, phosphodiesterases and phosphomonoesterases used in the first step of tris(2-chloroethyl) phosphate (TCEP) degradation^25–28^. However, no other enzymes have been implicated in OPE degradation and no other OPE-degrading organisms have been identified or isolated. Even though the P in OPEs can be used by some bacterial isolates, it remains unknown whether anthropogenic OPEs are actually degraded in natural waters.

The biodegradation of OPEs can have a direct impact on our understanding of the biogeochemical cycle of P in seawater. First, since large parts of the ocean have suboptimal concentrations of P, this could promote the use of OPEs as P sources. Secondly, OPEs could be modifying marine microbial communities, as a result of selective advantages of OPE-degraders in P-limited ecosystems. The targeted OPEs are a fraction of a larger, but still uncharacterized and unquantified, pool of synthetic organic P. A priori, at the concentrations commonly found in the marine environment, it is unlikely that the OPEs exert a toxicological effect on bacteria on their own, even though they could contribute to the overall effects of the mixture of synthetic chemicals present in seawater^29^.

We designed a set of experiments to study the microbial responses to OPE additions at environmentally relevant concentrations in P-limited seawater. The experiments were performed using waters from the Blanes Bay and Barcelona coast (both in the NW Mediterranean Sea) during the summer period, normally under P-limitation^30^. In these experiments, OPEs degradation rates were quantified, concurrently with the physiological response and the changes in taxonomic composition of the prokaryotic communities.

## Results and Discussion

### Is there consumption of OPEs in seawater?

**Figure 1:**
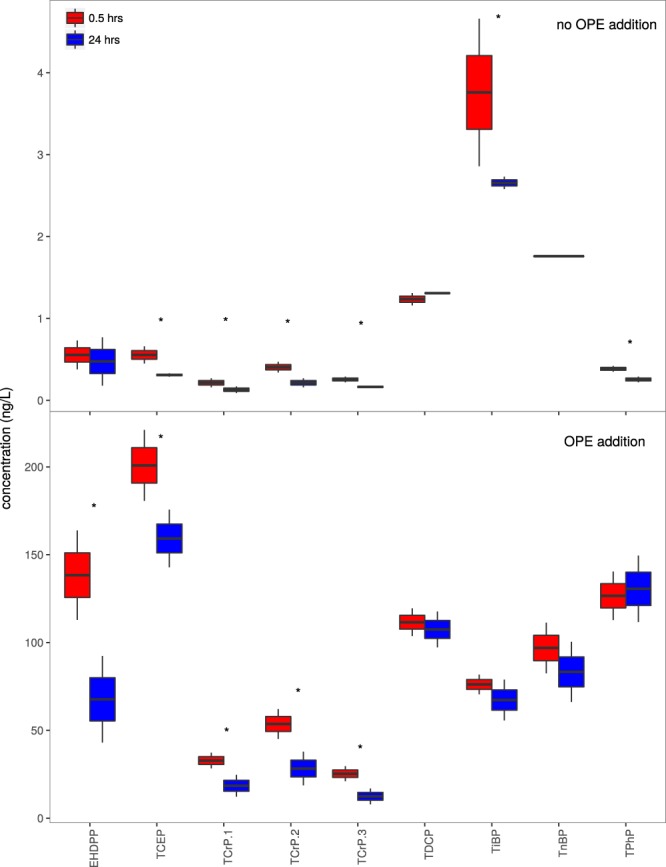
Concentrations of the measured organophosphate esters (OPEs) in unamended seawater (no OPE additions, upper panel) and in the OPE addition treatments (200 ng/L of a mixture of several OPE compounds, lower panel) after 0.5 hrs and 24 hrs. Compounds with significant different concentration between times points are labeled with an asterisk (t-test, P < 0.05). EHDPP: 2-ethylhexyl diphenyl phosphate; TCEP: tris(2-chloroethyl) phosphate; TCrP: tricresyl phosphate (4 isomers); TDCP: tris[2-chloro-1-(chloromethyl)ethyl] phosphate, TiBP: triisobutyl phosphate, TnBP: tri-n-butyl phosphate, TPhP: triphenyl phosphate.

 Bacterial production significantly increased in the controls of Blanes Bay experiment (with no OPE addition) when glucose (C) and inorganic phosphorus (P) was added to the seawater, but no when only C was added (Fig. S1), strongly suggesting that the waters were P-limited as it has been observed in the summer period in the same sampling site^30^. In order to assess whether OPEs are degraded in seawater, variations in OPE concentrations were determined after 24 hours of incubation, with a concurrent identification of the prokaryotic cells reacting to the OPEs (Table S1). The sum of the OPEs concentrations averaged 10.5 ± 1.0 ng/L in the controls at the initial time and decreased to 7.0 ± 0.7 ng/L after 24 hours of incubation (Fig. 1 and Table 1). The chlorinated OPEs, TCEP and TDCP, accounted for a minor proportion (16.1 ± 2.6%) of total OPEs. The most abundant OPE was TiBP (3.7 ± 1.3 ng/L) followed by TCrP (sum of three isomers) (2.9 ± ng/L). The less abundant targeted OPE was TPhP (0.4 ± 0.1 ng/L). The initial concentrations in controls account for the OPE occurrence in coastal seawaters from Blanes Bay in the NW Mediterranean sea. While OPE concentrations have been reported previously for the Mediterranean atmosphere^12^, to the best of our knowledge, this is the first report of OPEs occurrence in Mediterranean waters. The concentrations of TCEP, TiBP, TnBP, TDCP, and TPhP in coastal Mediterranean are 2–20 times higher than those reported for the North Atlantic and Arctic open oceans^17^, but similar to or up to 10 times lower (depending on the compound) than those reported in the Bohai and Yellow seas^20^. The concentrations of OPEs in the treatments were obviously higher (Fig. 1) as the compounds were added at concentrations above the expected levels for the Mediterranean Sea.

**Table 1:** First order reaction constants and half-life times of the analyzed OPE compounds.

	coefficient	SE	E-folding (−1/k)	Half-life time (hours)	Adjusted R-squared	p-value
TiBP	−0.0095	0.0058	105.3	73.0	0.19	0.16
TnBP	−0.0092	0.0066	108.7	75.3	0.12	0.21
**TCEP**	**−0.0166**	0.0059	60.2	41.8	0.49	**0.03**
TDCP	−0.0140	0.0163	71.4	49.5	−0.04	0.42
TPhP	−0.0071	0.0065	140.8	97.6	0.03	0.32
EHDPP	−0.0227	0.0157	44.1	30.5	0.13	0.20
TEHP	−0.0132	0.0094	75.8	52.5	0.12	0.21
**TCrP-1**	**−0.0238**	0.0096	42.0	29.1	0.43	**0.05**
**TCrP-2**	**−0.0275**	0.0084	36.4	25.2	0.58	**0.02**
**TCrP-3**	**−0.0252**	0.0085	39.7	27.5	0.53	**0.03**

Rate constants that were significantly different than zero are highlighted in bold (p < 0.05). EHDPP: 2-ethylhexyl diphenyl phosphate; TCEP: tris(2-chloroethyl) phosphate; TCrP: tricresyl phosphate (4 isomers); TDCP: tris[2-chloro-1-(chloromethyl)ethyl] phosphate, TiBP: triisobutyl phosphate, TnBP: tri-n-butyl phosphate, TPhP: triphenyl phosphate. E-folding and half-life times are the time needed to decrease the concentration to 1/e, or to half, the initial concentrations respectively.

The average concentration of inorganic P and dissolved organic carbon (DOC) at Blanes bay during summer is 0.1 µM and 55 µM^31,32^. Therefore, OPE concentrations accounted for 0.01% (±0.03%) of inorganic P, calculated in PO_4_^3−^ units in seawater (controls), and 0.31% (±0.15%) in OPE enrichments. In terms of DOC, OPE concentrations accounted for 0.003% (±0.007%) of DOC in the controls and 0.052% (±0.023%) in the enrichments. Despite OPEs accounting for such a low fraction of the Pi and DOC present in seawater, most analyzed OPEs decreased after 24 hours in both controls and OPE amendments (Fig. 1, Table 1). The rate constants for the decrease of concentrations were estimated assuming a first order reaction (see methods), and half lived estimated from the rate constants (Table 1). The decrease in concentrations was statistically significant for TiBP, EHDPP, TCEP and 3 isomers of TCrP (t-test, p < 0.05, Fig. 1). The estimated degradation rate constants (considering both controls and OPEs amendments) were significantly different than zero for TCEP and 3 isomers of TCrP. The predicted half-lives of these compounds ranged from 25 to 73 hours (Table 1). Although abiotic hydrolysis has been described for OPEs at basic pH^33^, the degradation rates reported for hydrolysis at marine pH suggest this degradation is negligible for a period of 24 hours. In addition, the experiments were performed in the dark to prevent photodegradation. Other potential mechanisms driving a decrease of concentrations would be sorption to bottle walls, that if occurred, it was at the beginning of the experiment when OPEs were spiked in the bottles and the solvent let to evaporate. Uptake of hydrophobic organic compounds is thought to be a passive diffusive uptake, only, as no active transport through the membrane has been reported for OPEs or other hydrophobic synthetic chemicals. With the octanol-water partition coefficients for the targeted OPEs (log K_OW_ ranging from 1.6 to 6.3) and the bacterial abundances in the incubations, the decrease due to passive uptake into cells would be lower than 1%. A hypothetical active uptake of OPEs by bacteria cannot be evaluated here, as it has never been reported and thus the extend and kinetics of this process remain unknown. In any case, uptake by bacteria is not contradictory with bacterial degradation. Therefore, the bulk of decrease in concentrations should be uniquely due to microbial degradation. An interesting point is whether there was degradation of the organic structure (Table S1) or microbial hydrolysis of P. The latter would release bioavailable P. n-alkanes and simple aliphatic groups are easier to degrade microbially than chlorinated paraffins and alkylated aromatic groups, and thus one could expect that the organic moiety of TnBP or TPhP be prone to be efficiently degraded. However, TnBP and TPhP showed longer half-lives than TCEP and TCrP (Table 1). This suggests that degradation preferentially occurred by hydrolysis of P, even though a confirmation of this cannot be given, as the degradation products were not analyzed.

Most of the previous assessments of microbial degradation of organophosphorus pollutants have been restricted to degradation of organophosphorus pesticides and chemical warfare agents (aryldialkyl phosphate)^34^, and there is a dearth of knowledge on bacterial degradation of trialkyl, triaryl and trihaloalkyl phosphate triesters, including OPE plasticizers and flame retardants^23^. OPEs utilization by isolated bacteria, yet mostly from soils, has been reported in the literature^8,23,24^. Interestingly, trihaloalkyl phosphate (like TCEP) and triaryl phosphate (like TCrP), which were significantly degraded in our incubations, have been reported to allow a moderate growth of two strains of Alphaproteobacteria using these two OPEs as a sole source of P, whereas trialkyl phosphate allowed only a slight growth of one of the strains^23,24,35^.

**Figure 2:**
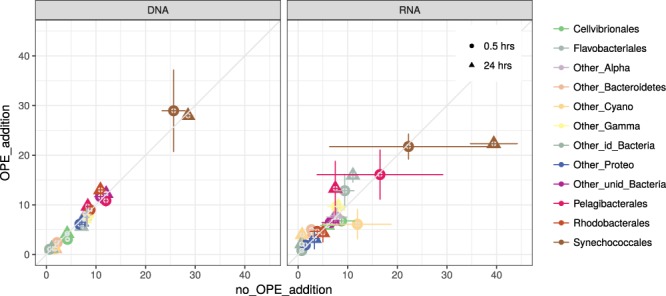
Contribution of the main phylogenetic groups classified at order level in the controls with no OPE addition (x axis) and in the OPE addition treatments (amendment with 200 ng/L final nominal concentration, y axis) to the total abundance (% total 16S rDNA, left panel) and the potential activity (% total rRNA reads, right panel). The values are the average of two replicates. Error bars indicate standard deviation.

In order to identify the members of the microbial community responsible for the observed OPEs degradation, we investigated changes in the diversity (rDNA) and potential activity (rRNA) of prokaryotes by sequencing the 16S rRNA gene and its transcripts. The initial prokaryotic community was dominated by the cyanobacteria *Synechococcus* (25.7 ± 3.3%), the Alphaproteobacteria SAR11 (12 ± 0.4%), followed by the Alphaproteobacteria Rhodobacterales (8.8 ± 0.2%), other Alphaproteobacteria (8.2 ± 0.1%) and the Bacteroidetes Flavobacteriales (8.1 ± 0.8%). In the controls without OPE additions, the composition remained fairly constant after 24 hours, and the only significant change detected was a decrease in SAR11 relative abundances (to 8.4 ± 0.4%) (Fig. 2). In OPE-amended treatments, Flavobacteriales significantly decreased their relative abundance (to 5.7 ± 0.5%) compared to controls (p < 0.05).

The quantification of rRNA allowed to assess the degree of activity of the different groups of bacteria^36^. In contrast to the results found for the rDNA; the relative activity (as abundance in 16S rRNA libraries) of Flavobacteriales significantly increased after 24 hours in the OPE enrichments (from 9.4 ± 2.6 to 15.9 ± 2.0%) (Fig. 2). SAR11 also increased their relative abundance in the 16S rRNA pool (from 7.5 ± 0.7% in the controls to 13.3 ± 7.7% in the OPEs treatments) after 24 hours (Fig. 2). Synechococcus showed an opposite pattern, dropping their relative activity from 39.5 ± 6.7% to 22.3 ± 0.7% after incubation with OPEs. The potential cytotoxic effect of OPEs on *Synechococcus* metabolism could have favored activities of the heterotrophic bacteria in the community.

The assessment of activity for individual taxa shows that there was a heterogeneity in the response within the taxonomical groups. Some taxa were up to 4-fold more abundant in the OPE treatments compared to the controls and vice versa (Table 2). Most taxa that increased after OPE additions were Flavobacteria and some alphaproteobacteria groups such as Pelagibacters (SAR11) (Table 2). This is consistent with previous reports that described the capacity of Flavobacteria and alphaproteobacteria members to degrade other organophosphorus compounds and OPEs respectively^8,22–24^. Interestingly, the first report of isolation of OP-degrading bacteria from soils was a *Flavobacterium* sp.^35^, and phosphotriesterases, the group of enzymes that can degrade organophosphorus compounds, were initially purified from *Flavobacterium* spp. isolates^5^. Individual OTUs belonging to other groups also responded to OPE additions. For example, within Alphaproteobacteria, up to 84 taxa responded positively to OPEs addition while 116 taxa decreased their relative activity (Table 2). In agreement, isolates from soils and sediments belonging to the order Sphingomonadales, within the Alphaproteobacteria class, have been reported to be able to grow using TDCPP and TCEP as sole P source^23,24^. Unfortunately, up to 99% of marine bacteria are not amenable to be cultured^37^, leading to a lack of knowledge on the identity of bacteria degrading OPEs-triesters flame retardant and plasticizers in seawater. Takahashi *et al*. (2012a,b)^22,38^ were able to identify but not isolate members of *Acidovorax* and *Aquabacterium* as potential TDCPP and TCEP users, but these Betaproteobacteria have minor representation in seawater. Phosphotriesterase encoding genes, responsible for the first step of OPEs degradation, have been detected in several phylogenetic groups, including Gammaproteobacteria and Bacteroidetes^7^ and activity has been measured in phosphate triesters-degrading marine isolates belonging to Alphaproteobacteria^39^. Our data support this view of a wider range of OPE-responding bacteria in seawater.

**Table 2:** Number of taxonomically-identified 16S sequences that resulted in at least 4-fold enrichment (UP) or depletion (DOWN) in the OPEs amendments versus the non OPE addition controls in pairwise comparisons after 24 hrs of OPEs addition (24K24T).

Phylum	Class	Order	cDNA		DNA	
			Up	Down	Up	Down
Bacteroidetes	Favobacteriia	Flavobacteriales	49	39	29	52
	Saprospiria	Saprospirales	4	3	2	8
		Other_Bacteroidetes	22	22	9	27
Proteobacteria	Alphaproteobacteria	Pelagibacterales (SAR11)	17	25	32	55
		Rhodobacterales	16	13	14	19
		Rickettsiales	4	2	0	0
		Rhodospirillales	3	7	2	3
		Other_Alpha	44	69	23	81
	Gammaproteobacteria	Cellvibrionales	22	27	29	21
		Alteromonadales	6	1	0	2
		Other_Gamma	27	26	17	26
	Other_Proteo		15	23	17	26
Cyanobacteria		Synechococcales	1	24	5	12
		Cyano	20	1	22	41
Balneolaeota	Balneolia	Balneolales	4	0	2	2
		Other_identif_Bacteria	13	14	9	13
		Unid_Bacteria	34	47	37	59

Analysis repeated for the cDNA (activity) and DNA (abundance) libraries using EdgeR.

### How OPEs addition affects physiological traits of marine microbial communities?

We designed an experiment in which we created different trophic conditions to analyze the metabolic effects of OPEs addition to microbial communities. The experiment evaluated 3 trophic regimes: addition of labile C to stimulate microbial activities, addition of labile C and Pi to stimulate the community and avoid P limitation, and no addition of nutrients. Each trophic condition was assessed under 3 different treatments: no additions of OPEs (controls), and additions of low (environmentally relevant) and high concentrations of OPEs (see material and methods for details). Responses at the physiological level were identified by measuring prokaryotic abundances by flow cytometry, bacterial activity, the number of highly respiring cells (CTC), and the proportion of “dead or damaged cells” (after the NADS protocol).

**Figure 3:**
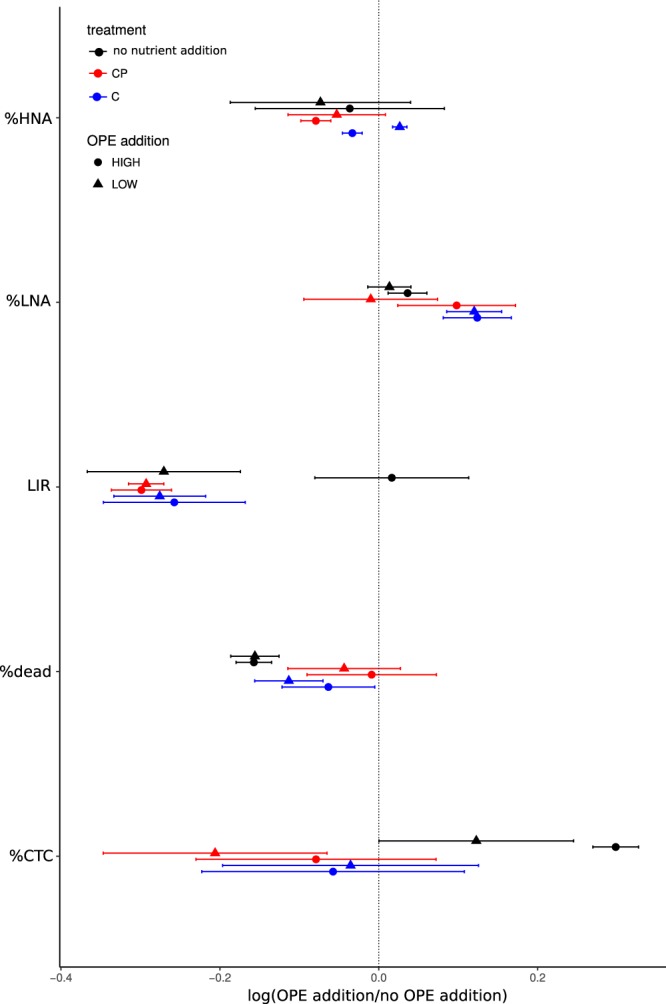
Effects of OPEs additions on microbial abundance and physiological parameters, expressed as ratio of values observed in the OPE addition treatments divided by values in the no addition controls when low (+200 ng/L final conc, triangles) or high (+2000 ng/L final conc., circles) OPEs were added in different assays: with no nutrient addition (black), with additions of glucose (C, blue) and with additions of glucose + phosphorus (CP, red). The values are the ratio of the average of two replicates. Error bars are calculated by error propagation. %HNA = percentage of high DNA prokaryotic cell abundance; %LNA = percentage of low DNA prokaryotic cell abundance; LIR: leucine incorporation rates as a proxy of bacterial activity; %dead: percentage of “dead/damaged” cells (%NADS); CTC: % of actively-respiring cells (CTC positive).

When no extra source of C and inorganic P was added, the percentage of damaged/dead cells (NADS) was lower in OPE treatments than in the controls, while the number of respiring cells (CTC+) was higher in the OPE treatments (Figs 3 and S2). Bacterial activity as leucine incorporation significantly increased after high addition of OPEs 24 hours of incubation, but was also significantly higher after low addition of OPEs for longer incubation times (48 hours, Figs 3 and S2). In contrast, these trends were not observed when the communities were stimulated using C and P (Figs 3 and S2). This suggests that OPE addition stimulate growth under limiting conditions when microbial communities are more prone to use these compounds as a source of C and/or P. However, OPEs do not appear to be preferred C and P sources.

Prokaryotes can be distinguished by their DNA content and cell size in the cytograms^40^, and cells are known to cluster in at least two groups according to whether they have low nucleic (LNA) or high nucleic acids (HNA) content. In general, bacterial growth was stimulated in LNA bacterial populations (Fig. 3). Abundances of LNA cells correlated with the relative abundances of SAR11, Rhodobacterales and Balneolales (Table 3), that also correlated with leucine incorporation and CTC counts in some cases (Table 3). Studies using flow cytometric cell sorting followed by high-throughput sequencing of 16S rRNA genes have shown different phylogenetic affiliation of LNA and HNA bacteria^41^. These studies suggest that these distinctive cytogram signatures can also reflect different life styles, HNA would reflect copiotroph bacteria such as Rhodobacterales and LNA frugal bacteria such as Pelagibacterales (mostly SAR11). The stimulation of LNA bacterial growth by OPE addition further supports the hypothesis that OPEs can be used as a P and/or C source, but seems to occur only for those groups that require low amounts of P. Thus, abundant groups in oceans such SAR11 would benefit from increased loads of OPEs in seawater. Flavobacteriales activity increased when exposed to higher OPE concentrations (see above), but their relative abundance significantly decreased after 24 hrs (Fig. 2), consistent with the fact that they are mostly composed of HNA bacteria^41^, and suggesting a complex interplay between some functional activities and abundance (growth).

**Table 3:** Significant Spearman correlations between concentration of pollutants, physiological indicators and relative abundances in 16S rRNA and rDNA libraries of selected taxonomical groups (N = 8).

		Concentration of OPEs									Physiological data		
		TiBP	TnBP	TCEP	TDCP	TPhP	EHDPP	TCrP1	TCrP2	TCrP3	LIR	FC_LOW	CTC
		ng/L	ng/L	ng/L	ng/L	ng/L	ng/L	ng/L	ng/L	ng/L	pmol Leu L^−1^h^−1^	cell/ml	cell/ml
cDNA Flavobacteriales	% 16S rRNA										*		
cDNA Other_Bacteroidetes	% 16S rRNA	**	*	**		*	**	**	**	*			
DNA Pelagibacterales(SAR11)	% 16S rDNA										*	**	
DNA Rhodobacterales	% 16S rDNA										*	*	*
DNA Other_Bacteroidetes	% 16S rDNA										*		
DNA Balneolales	% 16S rDNA											**	*
DNA Other Gamma	% 16S rDNA										*		

P values < 0.05 are labeled with * and < 0.01 with **. LIR: Leucine incorporation rates as a proxy of bacterial production, FC_LOW: low DNA prokaryotic cell abundance measured by flow cytometry. CTC: cell abundance of actively-respiring cells.

### Are OPEs used as P source in seawater?

**Figure 4:**
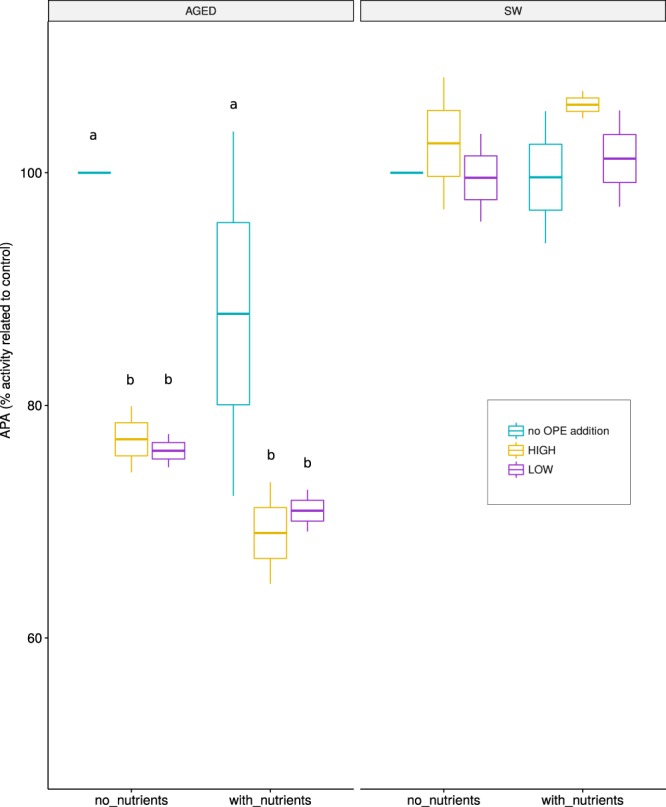
Alkaline phosphatase activity (APA, rates over 2.5 hours and scaled to the control with no carbon (C), no nitrogen (N) and no OPEs addition values) in 3 amendments (no OPE addition: control, LOW: addition of 100 ng/L of an OPEs mixture, HIGH: addition of 1000 ng/L of the OPEs mixture) using 2 different waters (SW: seawater, AGED: mixture of seawater and aged seawater (60:40)) under 2 conditions (with no C and N addition and with addition of glucose and ammonium). Error bars result from duplicate samples. Significant differences (p < 0.05) of mean values were analyzed using 2-way anova followed by a post-hoc Tukey HSD test and are labeled as different letters in the graph.

A universal response of microbial communities when the availability of Pi is not sufficient to meet their requirements is to induce mechanisms to scavenge Pi or use alternative sources of P^42^. Key enzymes of this response are alkaline phosphatases, which are broad spectrum monophosphoesterases that enable the cells to access a myriad of phosphorus compounds^43^. In order to test if OPEs are used as a P source, we tested if alkaline phosphatase activity (APA) varied when the microbial communities were challenged with high and low concentrations of OPEs as compared to no OPEs addition controls. The rationale behind the experiment was that if OPEs were used as a P source, we would observe a decrease in APA, because the monophosphoesters released during OPEs’ degradation would compete for the active sites of the enzyme with the fluorogenic substrate employed to quantify APA^44^. Since some studies have posed that APA may as well be involved in C acquisition by heterotrophic bacteria^45^, we also performed additions of carbon (C) and nitrogen (N) to force P limitation and alleviate any potential C and N deficiency. Our results showed a significant decrease in APA in the “aged seawater” treatment (see methods) upon OPE additions, with the lowest APA values in OPEs treatments (Fig. 4). The decrease was also evident when both C and N were supplied, suggesting that OPEs were mostly used as a P source. In contrast, APA was not affected in the “fresh seawater” treatment, suggesting that the community was not Pi stressed at the time of the experiment. These results indicate that under P limiting conditions, OPEs are used as a source of P by marine prokaryotes.

Although in principle, alkaline phosphatases should not be able to hydrolyze the tri-phosphoester bonds present in OPEs structures, they have been shown to be needed in OPEs utilization^26^. For example, strains of *Shingobium* sp degrade TCEP and TDCPP by hydrolysis of the three phosphoester bonds, sequentially using a phosphotriesterase, a phosphodiesterase (PDE) and phosphomonoesterases (PME)^26,28^. These strains harbour the two alkaline phosphatase genes that are more widespread in marine bacteria, *phoX* and *phoA*^6^, and deletion of these two genes resulted in the complete loss of cell growth on medium containing TCEP as the sole phosphorus source^26^.

Phosphate esters represents >75% of the total dissolved organic phosphorus pool (DOP) in the marine environment^46,47^ and the capacity to degrade DOP compounds seems to be a widespread feature among marine bacteria^44^. Our study shows for the first time that a family of synthetic organophosphorus compounds, widely distributed in oceanic waters, can be used as a source of P under limiting conditions. The biogeochemical significance of this is difficult to evaluate with the current knowledge of the quantity and structure of synthetic organics containing P reaching the marine environment. Future research should aim at identifying a larger number of anthropogenic organophosphorus compounds in marine systems and use culture-independent techniques, such as metatranscriptomic analysis, to provide insights on the most common set of enzymes used to degrade OPE plasticizers and flame retardants.

## Material and Methods

### Description of Experiments

#### Degradation of OPEs in seawater and potential microorganisms involved

Unfiltered seawater from the Blanes Bay Microbial Observatory (BBMO, Mediterranean Sea 41°40′N, 2°48′E) was sampled on July 7^th^, 2014 from the surface (0.5 m depth) into pre-cleaned metallic carboys (20 L). Carboys were transported to the lab within 1 hour. A mix of 10 OPEs dissolved in acetone (compounds listed in Table 1, final nominal concentration 200 ng/L) was added to previously baked (450 °C, 4 hours) 2 L glass bottles, and the acetone was let to volatilize (OPEs treatment). Controls consisted on adding the same amount of acetone with no OPEs (Control treatment). The collected seawater was added to the 2 L glass bottles and incubated at *in situ* temperature in the dark for 48 hours. The experiment was run in duplicate. Samples for 16S rRNA and rDNA Illumina MiSeq library construction and for determination of OPEs concentrations were collected 0.5 and 24 hours after addition of seawater.

#### Physiological responses of microbial communities to OPEs addition

A second experiment was performed with the unfiltered water collected from Blanes Bay (July 7^th^, 2014). The experiment consisted on adding OPEs at two different concentrations (“LOW”: 200 ng/L as nominal OPEs concentration; “HIGH”: 2000 ng/L as nominal OPEs concentration) and a control without OPEs addition. Each of these treatments included three different trophic conditions: an unenriched control (“no_ nutrient addition”), one with glucose amendments (24 μM final concentration, “C”) to stimulate bacterial growth, and one enrichment with both glucose and phosphate (24 μM of glucose and 0.6 μM PO_4_ final concentration, “CP”) to stimulate bacterial growth and prevent any potential P limitation. Treatments and controls were incubated in duplicate during 24 hours in the dark at *in situ* temperature. Samples to quantify the percentage of damaged or dead cells (NADS, see below), the abundance of actively-respiring bacteria (CTC), the abundance of prokaryotic cells and bacterial activity were taken at the end of the incubation (see below for details).

#### Attenuation of P stress due to OPEs additions

Alkaline phosphatase activity (APA) is used as indicator of P-limitation in seawater^48^. This experiment was carried out on June 17^th^ 2014. Since Pi availability might not be limiting at this period of the year^30^, in this experiment we used both freshly unfiltered collected seawater from the Barcelona coast, and that same seawater diluted 60:40 with 0.2 µm-filtered “aged seawater”, i.e. seawater that had been collected 1 year before the experiment and had been kept in the dark to allow for consumption of most of the labile dissolved organic compounds (DOC) and nutrients^49^. For both the “fresh seawater” and the “aged seawater” we performed two treatments: an addition of carbon (C; glucose at 10 μM final concentration) and nitrogen (N; ammonium at 2 μM final concentration) to force bacterial growth to be limited by P, and another without any addition. Each of the mixtures were split into 6 amber baked-glass vials (60 ml): 2 received an addition of OPEs at low concentrations (100 ng/L, nominal concentration), 2 received OPEs at high concentration (1000 ng/L, nominal concentration), and 2 did not receive any OPE addition (control). APA was determined after 0, 1, 2.5, 4.5 and 24 hours of incubation (see below).

### Analysis of OPEs in seawater

Samples were analyzed for the following OPEs: tris(2-chloroethyl) phosphate (TCEP), tris[2-chloro-1-(chloromethyl)ethyl] phosphate (TDCP), tris(1-chloro-2-propyl) phosphate (TCPPs, 3 isomers), triisobutyl phosphate (TiBP), tri-n-butyl phosphate (TnBP), triphenyl phosphate (TPhP), 2-ethylhexyl diphenyl phosphate (EHDPP), tris(2-ethylhexyl) phosphate (TEHP), and tricresyl phosphate (TCrP, 4 isomers) (Table S1). Dissolved-phase OPE concentrations were determined after 30 minutes and after 24 hours of incubation. After cells had been removed by filtering 2 L onto a 0.2-μm pore-size filter, a surrogate standard mix containing two deuterated OPEs (TnBP-d27, TPhP-d15) was added to the filtered water. This water was then pre-concentrated on a solid-phase extraction 3 cc Bond Elut PPL (Agilent, 200 mg) containing 200 mg of sorbent, using a vacuum manifold. The PPL cartridges had been previously conditioned with 5 mL of hexane followed by 5 mL of dichloromethane:hexane (2:1), 5 mL of dichloromethane:acetone (1:1) and 5 mL of HPLC-grade water. The cartridges were eluted with 5 mL of hexane, 10 mL of dichloromethane:hexane (2:1), and 5 mL of dichloromethane:acetone (1:1). Any aqueous residual in the extract was purified on a glass funnel filled with 50–60 g of anhydrous sodium sulfate. The extract was concentrated to 50–100 μL under a gentle stream of nitrogen. Prior to injection, additional deuterated standards (tri-n-propyl-d21 phosphate, malathion-d7) were added to extracts as internal standards for quantification. OPEs were analyzed by high resolution gas chromatography - mass spectrometry (HRGC-MS). Results were blank corrected. Details on the analytical procedure and quality assurance/quality control are presented in the SI.

### Nucleic acid extraction, library preparation and taxonomical identification of 16S rDNA and rRNA amplicons

Samples for 16S rRNA and rDNA library construction were collected after 30 minutes and 24 hours of the beginning of the experiment. Seawater (2L) from the incubations were filtered onto 47-mm-diameter, 0.2-μm pore-size PTFE filters (Millipore, Billerica, MA) under low vacuum pressure using a baked-glass filter holder. PTFE have been shown to be optimal in order to minimize sampling time (preserving RNA), but allowing an optimum extraction efficiency of RNA/DNA^50^ After filtration, each filter was placed into lysis buffer (50 mM Tris HCl, 40 mM EDTA, 0.75 M Sucrose) at −80 °C to preserve nucleic acids until analysis. Samples were incubated with lysozyme, proteinase K and sodium dodecyl sulphate (SDS), and nucleic acids were extracted simultaneously with phenol/chloroform/isoamyl alcohol (25: 24: 1 vol: vol: vol) and with chloroform/isoamyl alcohol (24: 1, vol: vol) as previously described^51^. The resulting solution was concentrated to 200 μL using an Amicon Ultra 10-kDa filter unit (Millipore). Half of the volume was incubated with RNAseA for 1 hour at 37 °C to remove the RNA. The other half of the sample was treated with DNase using the TURBO DNA-free kit (Ambion, Austin, TX) to remove the DNA. cDNA was synthesized using a SuperScript III reverse transcription system (Invitrogen) with random hexamer primers. Partial bacterial 16S gene fragments of both DNA and cDNA were amplified using primers 515 f/926r plus adaptors for Illumina MiSeq sequencing^52^. The PCR reaction mixture was thermocycled at 95 °C for 3 minutes, 30 cycles at 95 °C for 45 s, 50 °C for 45 s, and 68 °C for 90 s, followed by a final extension of 5 min at 68 °C. PCR of the RNA subsamples after DNAse treatment and before the RT reaction were used to check for potential DNA contamination. PCR amplicon sizes were checked in tris-acetate-EDTA (TAE) agarose gels. Illumina MiSeq sequencing was conducted at the Pompeu Fabra University Sequencing Service. We ended with an average of 85545 ± 28681 sequences per sample after preprocessing. Sequences have been deposited in the European Nucleotide Archive (ENA) under accession numbers ERS2213964 to ERS2213995.

### Alkaline phosphatase activity (APA)

APA was determined in triplicate subsamples by monitoring the rate of hydrolysis of the fluorogenic substrate 4-methylumbelliferyl phosphate (MUP-P, Invitrogen, Eugene, OR, USA) at a final concentration of 10 µM along a time course of 24 hours. Activity rates were calculated from the slope of the linear part of the curve of fluorescence (rfu) against time^48^.

### Flow cytometric determination of prokaryotic cell abundances

Subsamples of 1.8 ml for quantification of abundances of prokaryotes were fixed with 1% buffered paraformaldehyde solution (pH 7.0) plus 0.05% glutaraldehyde, left at room temperature in the dark for 10 minutes, flash-frozen in liquid nitrogen and stored at −80 °C. Prokaryotic cell abundance was estimated by flow cytometry as described elsewhere^53^.

### Abundance of membrane-compromised cells (NADS) and actively-respiring bacteria (CTC) determined by flow cytometry

Two physiological probes to test the metabolic and physiological single-cell status of prokaryotic microorganisms were employed following described protocols^40,54^. Briefly, cells with intact (named “live” cells) versus cells with damaged membranes (named “dead” cells) were enumerated using the Nucleic-Acid-Double-Staining (NADS) viability protocol, which combines the membrane-permeable nucleic acid strain SybrGreen I (SG1, Molecular Probes, Eugene, OR) and the membrane impermeable propidium iodide (PI, Sigma Chemical Co.) fluorescent probes. We incubated freshly collected seawater samples with a mixture of SG1 (10x final concentration) and PI (10 μg ml^−1^) for 20 min in the dark at room temperature. NADS+ (“live” cells) and NADS- (“dead” cells) were enumerated by flow cytometry as described elsewhere^38^. The abundance of highly respiring bacteria was determined using the 5-cyano-2,3-ditolyl tetrazolium chloride probe (CTC, Polysciences), an indicator of strong respiratory activity with formation of a red-fluorescent formazan salt within the electron transport chain of respiration that is detectable by flow cytometry^55^. A fresh subsample was incubated with daily prepared CTC stock solutions at 5 mM for 90 minutes. The red fluorescence of CTC and side light scatter were used to discriminate the CTC positive cells from other particles and an orange to red fluorescence plot to exclude picoautotrophs in the flow cytometer.

### Bacterial activity (as Leucine Incorporation Rates (LIR))

BP was estimated as the incorporation of ^3^H-leucine into protein as reported elseshwere^56,57^. Briefly, 1.2 ml quadruplicate live and duplicate killed (5% trichloroacetic acid (TCA)) subsamples were incubated with ^3^H-leucine (40 nM) for 2 hours at *in situ* temperature in the dark. Incubations were stopped by addition of 120 μl of cold TCA 50% and then frozen (−20 °C) until further processing by centrifugation and TCA rinsing.

### Bioinformatics

Sequence reads were cleaned and merged with the DADA2 pipeline version 1.4.0^58^ as packaged in eemisdada2 (https://github.com/erikrikarddaniel/eemisdada2). To establish taxonomy, sequences were aligned to NCBI’s RefSeq RNA database (downloaded 16 August 2017) using the LAST aligner^59^, followed by classification with MEGAN version 6.8.18 (minimum score: 50)^60^.

### Statistical analyses

Significances (p < 0.05) of degradation rates different than zero were tested by least squares linear regression of ln(C_t_/C_0_) versus time, where C_t_ and C_0_ are the OPE concentrations at times t and initial, respectively. The slope of this regression line is the reaction rate constant (k, h^−1^). We assumed then a first order decay of concentrations, and thus the OPE concentrations from the experiments with OPE addition and no OPE addition were used together to derive k, as it is independent of concentration. Half-lives and e-folding times (Table 1) were estimated from k values. Cumulative distributions of all variables were compared against a normal-distribution function using the Shapiro–Wilk test. We used ANOVAs and Tukey’s HSD post-hoc test to determine whether variables exhibited significant variation between treatments. If only two groups were compared (for instance, low OPE amendment vs. control), a t-test was used. Spearman rank-order correlation coefficients were determined for pairwise comparisons of OPEs concentrations and biological variables. EdgeR package in R 3.2.1 (R Core Team 2017) was used to identify those OTUs with significantly different contribution to community structure in the different treatments.

## Electronic supplementary material

Supplementary Inofrmation

## Data Availability

Sequences have been deposited in the European Nucleotide Archive (ENA) under accession numbers ERS2213964 to ERS2213995. Additional datasets generated during and/or analysed during the current study are given in the supplementary material or available from the corresponding author on reasonable request.
