# Supplementary material for: Microbial consumption of organophosphate esters in seawater under phosphorus limited conditions
Source: Sci Rep. 2019 Jan 18;9:233. doi: 10.1038/s41598-018-36635-2 (PMC6338803; doi:10.1038/s41598-018-36635-2)
Supplement: Supplementary file 1 — Supplementary Inofrmation [file 41598_2018_36635_MOESM1_ESM.pdf]

Supplemental Information

Microbial consumption of organophosphate esters in seawater under phosphorus limited conditions

Maria Vila-Costa[1]*, Marta Sebastián[2,3], Mariana Pizarro[1], Elena Cerro-Gálvez[1], Daniel Lundin[4], Josep M. Gasol[2], Jordi Dachs.[1]

[1]Department of Environmental Chemistry, IDAEA-CSIC-Jordi Girona 18-26, Barcelona 08034, Barcelona, Catalunya, Spain.

[2]Departament de Biologia Marina i Oceanografia, Institut de Ciències del Mar, CSIC, Barcelona, Catalunya, Spain

[3]Instituto de Oceanografía y Cambio Global, IOCAG, Universidad de Las Palmas de Gran Canaria, 35214, Gran Canaria, Spain.

[4]Centre for Ecology and Evolution in Microbial Model Systems, EEMiS, Linnaeus University, Barlastgatan 11, 391 82 Kalmar, Sweden.

* corresponding author: mvcqam@cid.csic.es

Text S1: Quality assurance/Quality control for Analysis of OPEs

OPEs were analyzed from the water previously filtrated on 0.2-µm pore-size filters, which were used to collect the nucleic acids. We compared potential contamination from polycarbonate and Teflon filters (47-mm-diameter, 0.2-µm pore-size GTTP and PTFE filters, Millipore, Billerica, MA) without detecting significant differences. PTFE filters were selected as they allow fastest filtration times, and thus better conservation of nucleic acids[1].

The limits of quantification (LOQ) were determined as the average levels in the blanks plus three times the standard deviation of the levels in blanks. Procedural blanks were performed with surrogate spiked cartridges (Bond Elut PPL cartridges (Agilent, 200 mg, 3 ml) and with milliQ water. A procedural contamination of TCPP and TEHP was detected and these OPEs were removed from the list of targeted compounds. LOQ of the targeted compounds ranged from 0.004 ng for TCrP-1 to 4 ng for TiBP (median LOQ of 0.8 ng for the targeted compounds).

Matrix spikes were performed for evaluating the recoveries of the targeted OPEs. These matrix spikes consisted of HPLC grade water spiked with the mix of targeted OPEs. The analytical procedure was the same as for the controls and treatments of the experiments. Recoveries ranged from 45% for TiBP to 103% for TCEP (median of 77% for targeted OPES), and of 65 -74% for D27-TBP and D15-TphP used as surrogates.

Table S1. Chemical structure of the OPEs analyzed in this study. MW = Molecular Weight (g/mol)

Compound	Acronym	Formula	MW	Structure
tris(2-chloroethyl) phosphate	TCEP	$C_6H_{12}Cl_3O_4P$	285.49	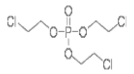
tris[2-chloro-1-(chloromethyl)ethyl] phosphate	TDCP	$C_9H_{15}Cl_6O_4P$	430.9	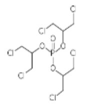
tris(1-chloro-2-propyl) phosphate (3 isomers)	TCPP	$C_9H_{18}Cl_3O_4P$	327.57	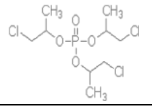
Tri-isobutyl phosphate	TiBP	$C_{12}H_{27}O_4P$	266.31	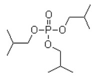
tri-n-butyl phosphate	TnBP	$C_{12}H_{27}O_4P$	266.31	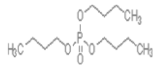
triphenyl phosphate	TPhP	$C_{18}H_{15}O_4P$	326.28	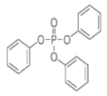
2-ethylhexyl diphenyl phosphate	EHDPP	$C_{24}H_{51}O_4P$	362.4	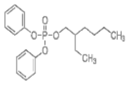
tris(2-ethylhexyl) phosphate	TEHP	$C_{20}H_{27}O_4P$	434.63	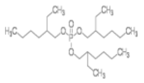
tricresyl phosphate (4 isomers)	TcrP	$C_{21}H_{21}O_4P$	368.36	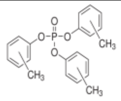

Figure S1. Leucine incorporation rates in Blanes Bay samples in the controls with no OPE additions (K), in low OPE addition treatment (LOW, +200 ng/L final conc) and in high OPE addition treatment (HIGH, +2000 ng/L final conc.) after no nutrient addition (blue), with additions of glucose (+C, orange) and with additions of glucose + phosphorus (+C+P, violet). The values are the ratio of the average of two replicates. Error bars are standard deviation of replicates. Significant differences ($p<0.05$) of mean values were analyzed using 1-way anova followed by a post-hoc Tukey HSD test and labeled in the graph.

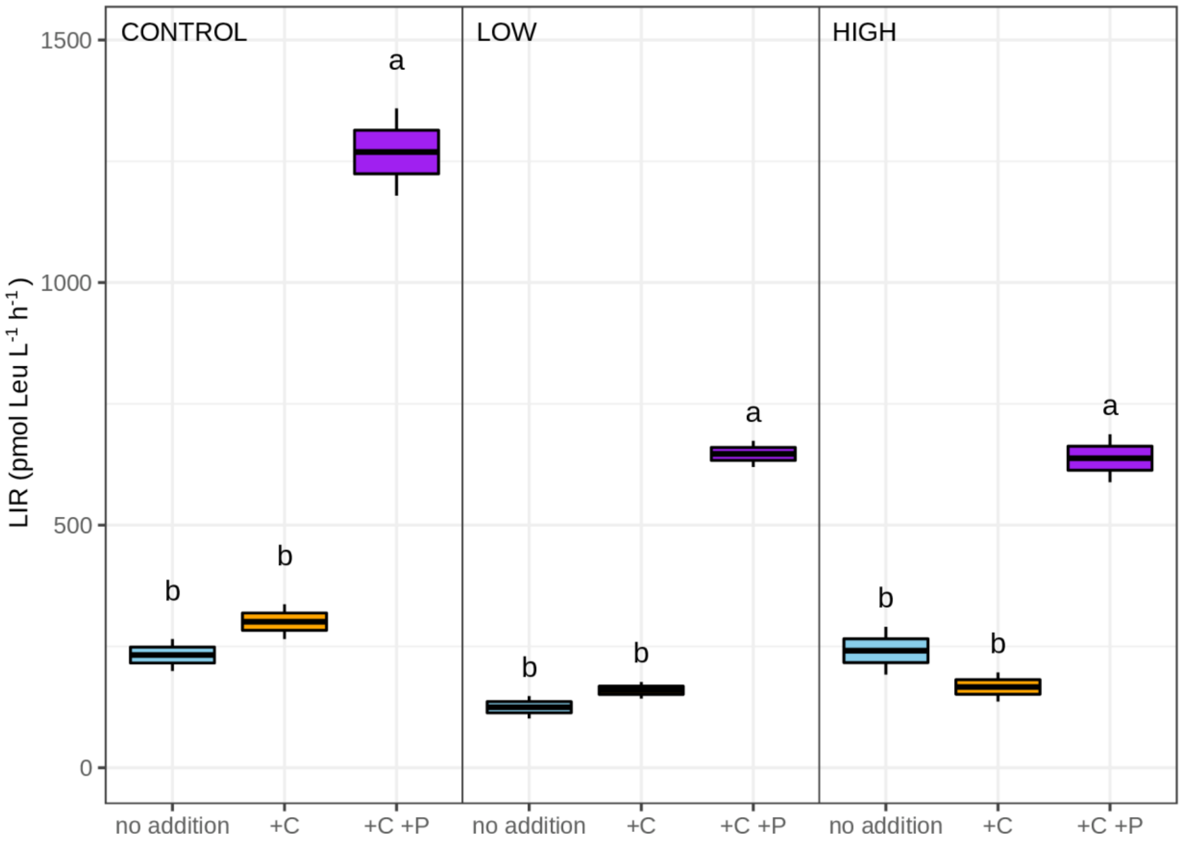

Figure S2. Effects of OPEs on microbial abundance and physiological parameters observed in the controls with no OPE additions (K), in low OPE addition treatment (LOW, +200 ng/L final conc) and in high OPE addition treatment (HIGH, +2000 ng/L final conc.) after no nutrient addition (blue), with additions of glucose (+C, orange) and with additions of glucose + phosphorus (CP, violet). The values are the ratio of the average of two replicates. Error bars are standard deviation of replicates. LNA = low DNA prokaryotic cell abundance; HNA = high DNA prokaryotic cell abundance; CTC: cell abundance of actively-respiring cells; %dead: percentage of "damaged/dead" cells (%NADS). Significant differences ($p<0.05$) of mean values were analyzed using 1-way anova followed by a post-hoc Tukey HSD test and labeled in the graph.

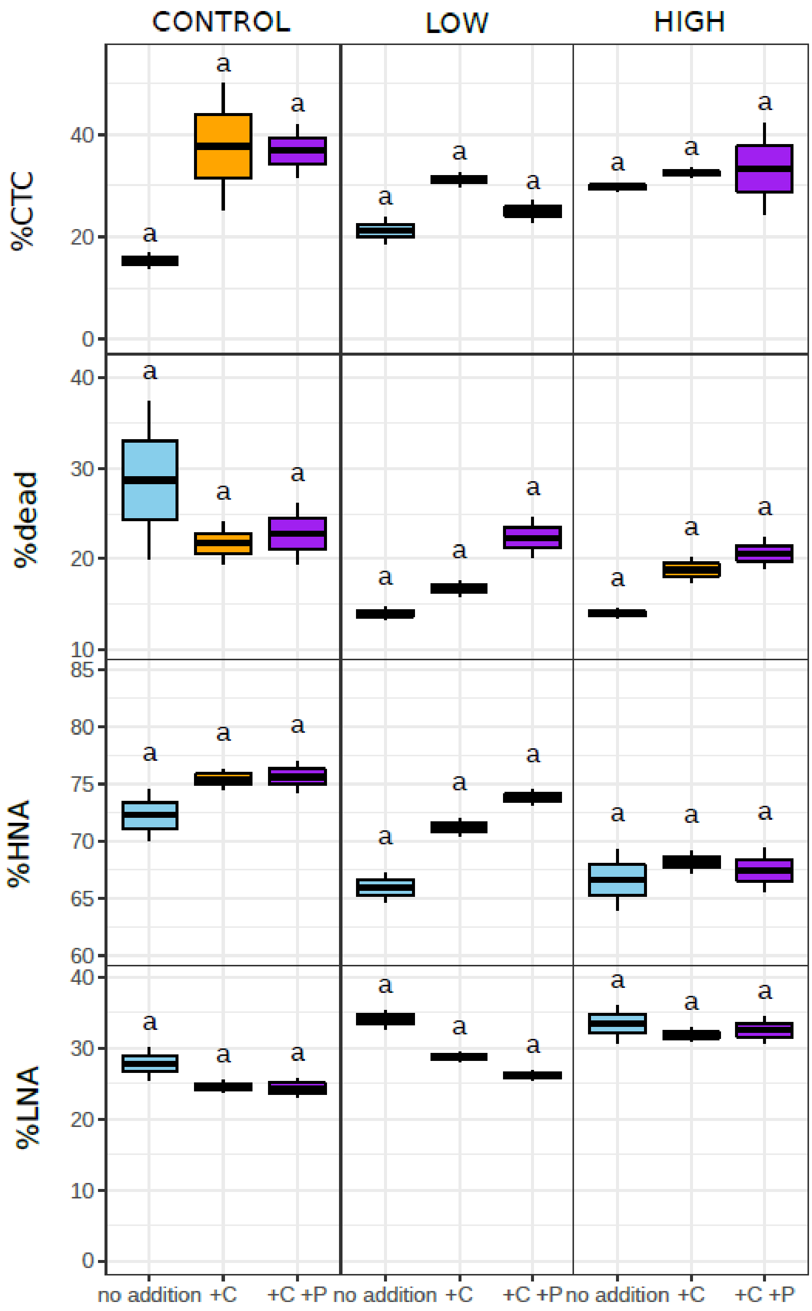

Figure S3. Effects of OPEs on microbial abundance and physiological parameters observed in the OPE addition treatments (LOW, +200 ng/L final nominal concentration), and in the no addition controls (K). These measurements were performed with water from the same bottles where OPEs concentrations and nucleic acids were extracted and measured (at time 48 hours). Error bars are standard deviation of replicates. LNA = low DNA prokaryotic cell abundance; HNA = high DNA prokaryotic cell abundance; CTC: cell abundance of actively-respiring cells; LIR: leucine incorporation rate as a proxy of bacterial production; %dead: percentage of “damaged/dead” cells (%NADS).

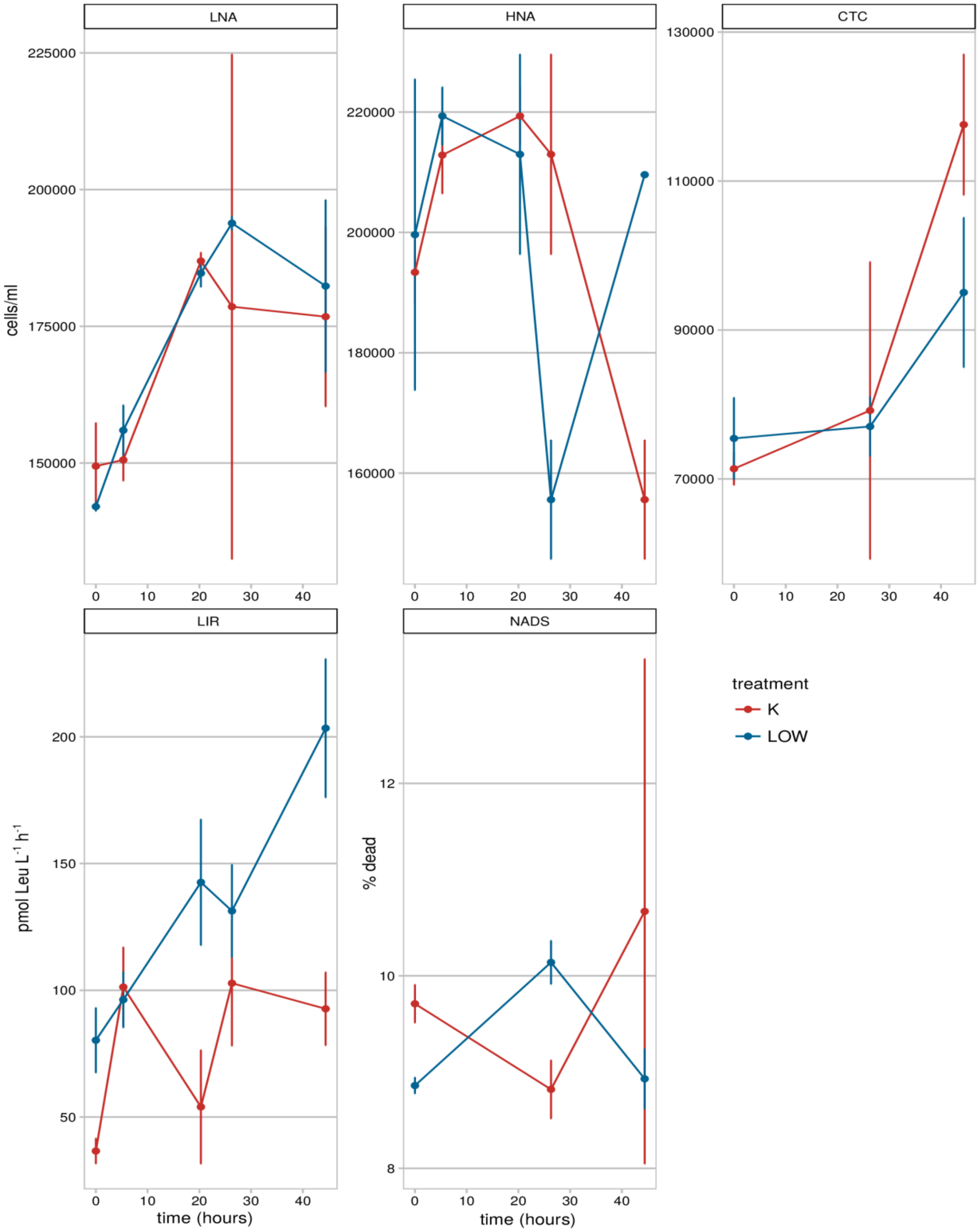

References

(1) Fernández-Pinos, M. C.; Casado, M.; Caballero, G.; Zinser, E. R.; Dachs, J.; Piña, B. Clade-specific quantitative analysis of photosynthetic gene expression in prochlorococcus. *PLoS One* **2015**, *10* (8)
